# Insights into Muscle Degeneration from Heritable Inclusion Body Myopathies

**DOI:** 10.3389/fnagi.2015.00013

**Published:** 2015-02-12

**Authors:** Sabine Krause

**Affiliations:** ^1^Laboratory for Molecular Myology, Department of Neurology, Friedrich Baur Institute, Ludwig Maximilians University, Munich, Germany

**Keywords:** VCP/p97, IBMPFD, GNE myopathy, multisystem proteinopathy, HNRNPA1

## Abstract

Muscle mass and function are gradually lost in age-related, degenerative neuromuscular disorders, which also reflect the clinical hallmarks of sarcopenia. The consensus definition of sarcopenia includes a condition of age-related loss of muscle mass, quality, and strength. The most common acquired muscle disease affecting adults aged over 50 years is sporadic inclusion body myositis (sIBM). Besides inflammatory effects and immune-mediated muscle injury, degenerative myofiber changes are characteristic features of the disease. Although the earliest triggering events in sIBM remain elusive, a plethora of downstream mechanisms are implicated in the pathophysiology of muscle wasting. Although it remains controversial whether hereditary forms of inclusion body myopathy (IBM) may be considered as degenerative sIBM disease models, partial pathophysiological aspects can mimic the much more frequent sporadic condition, in particular the occurrence of inclusion bodies in skeletal muscle. Various clinical aspects in genetically determined skeletal muscle disorders reflect age-related alterations observed in sarcopenia. Several intriguing clues from monogenic defects in heritable IBMs contributing to the molecular basis of muscle loss will be discussed with special emphasis on inclusion body myopathy with Paget’s disease of bone and frontotemporal dementia (IBMPFD) and GNE myopathy. Finally, also the recently identified dominant multisystem proteinopathy will be considered, which may rarely present as IBM.

## Introduction

Pathogenic mutations in genes involved in diverse cellular pathways as VCP/p97, also called valosin-containing protein [resulting in autosomal-dominant inclusion body myopathy (IBM) associated with Paget’s disease of bone and frontotemporal dementia, IBMPFD] and *GNE*, also referred to as UDP-*N*-acetyl-d-glucosamine 2-epimerase/*N*-acetylmannosamine kinase (GNE) (resulting in biallelic GNE myopathy) can elicit a pathophysiological phenotype in skeletal muscle that partially overlaps with histopathology findings in sporadic inclusion body myositis (sIBM), in particular the detection of inclusion bodies in skeletal muscle. While formal proof is lacking, it remains a hypothesis that genetically determined IBM syndromes may share some aspects of the pathogenetic cascade.

However, immune-mediated, inflammatory changes are a hallmark of sIBM and only a very rare exception in GNE myopathy (Krause et al., 2003; Yabe et al., 2003). Furthermore, the typical clinical presentation of sIBM features progressive muscle weakness in the knee extensors and the long finger flexors (Rose and ENMC IBM Working Group, 2013). By contrast, in GNE myopathy the quadriceps muscles are remarkably spared from muscle weakness (reviewed in Nishino et al., 2014). IBMPFD patients present clinically with a variable pattern of slowly progressive proximal and distal pareses (reviewed in Weihl et al., 2009). Scapular winging in IBMPFD may be as prominent as in neuromuscular shoulder girdle or limb girdle syndromes (Kimonis et al., 2008; Stojkovic et al., 2009).

VCP/p97 acts as an ubiquitin-selective multitasking switchboard in regulating basic cellular proteostasis including autophagy and myosin assembly in skeletal muscle (reviewed by Pokrzywa and Hoppe, 2013; Meyer and Weihl, 2014). GNE, the key enzyme of sialic acid biosynthesis, can regulate muscle glycoprotein sialylation and might contribute to additional cellular signaling pathways (reviewed by Nishino et al., 2014). *HNRNPA1*, the ubiquitously expressed gene for the heterogeneous nuclear ribonucleoprotein A1 is the causative gene for the rare multisystem proteinopathy, which may be associated with IBM was identified only very recently (Kim et al., 2013a).

In the adult-onset, heritable degenerative neuromuscular disorders, muscle mass and function are gradually lost and reflect the clinical hallmarks of sarcopenia. The consensus definition of sarcopenia includes a condition of age-related loss of muscle mass, quality, and strength (Cruz-Jentoft et al., 2010; Fielding et al., 2011). Key findings potentially related to sarcopenia at multiple levels of muscle metabolism in hereditary IBMs like IBMPFD and GNE myopathy as well as the HNRNPA1-associated multisystem proteinopathy presenting as IBM will be highlighted.

## IBMPFD Associated with VCP/p97 Mutations

The multifaceted role of the AAA-family ATPase VCP/p97 in the pathophysiology of IBMPFD involves numerous essential signaling pathways governing cellular homeostasis and depends on the cellular context. To exploit the therapeutic potential of VCP/p97, it is important to understand how its activity is regulated and how specific organs and cells can be targeted to regulate the versatile enzyme. The specificity of VCP/p97 depends significantly on cofactors, which confer precise catalytic function. The majority of pathogenic mutations in VCP/p97 resulting in neuromuscular disorders are localized in the cofactor binding domain (Tang et al., 2010) and abrogate binding to a distinct subset of cofactors (Fernandez-Saiz and Buchberger, 2010).

Myosin filament assembly is disturbed in myogenic cells from IBMPFD patients *in vitro* (Janiesch et al., 2007). Functional development of striated muscle depends on the accurate organization of regulatory, structural, and motor proteins into basic contractile elements called the sarcomeres. VCP/p97 acts in a ternary complex together with the cofactors CHIP and UFD2a to tightly regulate the level of the myosin-directed chaperone Unc45 within confined limits, which allow proper myosin filament assembly and sarcomere formation (Janiesch et al., 2007). CHIP is also called carboxyl terminus of Hsp70-interacting protein. UFD2a is the human homolog of yeast UFD2a, also known as ubiquitination factor E4B (UBE4B) and human Unc45 is the homolog of the *C. elegans* Unc45 chaperone, which is essential to regulate myosin-directed functions from fungi to vertebrates (reviewed in Hellerschmied and Clausen, 2014). Defective myosin assembly may contribute to myofiber fragility and reduced mechanical stability. Inclusion bodies might develop secondary and due high amounts of accumulated, unassembled myosin in the sarcoplasm. Similarly, in another form of familial IBM (IBM 3), dominantly inherited mutations in the head domain of MYH2 (adult skeletal muscle myosin heavy chain 2, fast myosin IIa) render MYH2 filament proteins to aggregate (Martinsson et al., 2000; Tajsharghi et al., 2005). Deregulated, elevated levels of Unc45 in skeletal muscle of affected IBMPFD patients suggest a related, relevant myosin biosynthesis defect *in vivo*.

Furthermore, deregulation of major protein degradation pathways has been implicated in VCP/p97-related disorders (Ju et al., 2009a,b; Tresse et al., 2010). An imbalance in protein turnover contributes also to muscle loss in sarcopenia (Barns et al., 2014).

Primary human myoblasts containing disease-related VCP/p97 mutations revealed increased apoptosis and defective maturation to myotubes *in vitro* (Vesa et al., 2009). This suggests that IBMPFD satellite cells have a reduced regeneration capability, and may generate defective myotubes thus contributing to muscle degeneration.

Furthermore, VCP/p97 is essential for mitochondrial quality control by PINK1/Parkin (PTEN induced putative kinase 1), which is associated with autosomal-recessive early-onset Parkinson disease. PINK1 prevents stress-induced mitochondrial dysfunction. This important protective capacity is impaired by disease-related VCP/p97 mutations (Kim et al., 2013b). Mutant VCP/p97 malfunction involves recruitment to and clearance of damaged mitochondria. These processes are paralleled in part by the mitochondrial theory of aging, which predicts the accumulation of damage by reactive oxygen species (ROS) over time to lead to age-associated mitochondrial impairment (Cesari et al., 2012; Johnson et al., 2013).

Epigenetic changes have been implicated in sarcopenia; however, detailed evidence is limited (Ong and Holbrook, 2014). For efficient gene expression, dynamic cycles of monoubiquitylation and de-ubiquitylation are indispensable (Wyce et al., 2007). In a collaborative project, we defined a novel regulatory role of VCP/p97 in histone H2B metabolism, which is conserved from yeast to man. Moreover, in IBMPFD cells carrying a point mutation at the highly conserved residue R155H that does not affect the ATPase activity, H2B de-ubiquitylation was significantly delayed 48 h after induction of differentiation *in vitro*. Our findings further extend the functional spectrum of VCP/p97 and suggest an additional molecular pathomechanism for IBMPFD at the level of chromatin remodeling and transcription control (Bonizec et al., 2015).

## GNE Myopathy

GNE myopathy is an inherited autosomal-recessive IBM. The causative gene was identified more than a decade ago (Eisenberg et al., 2001). The bi-functional enzyme UDP-*N*-acetyl-d-glucosamine 2-epimerase/*N*-acetylmannosamine kinase GNE is the key enzyme of the sialic acid biosynthesis pathway (Hinderlich et al., 1997; Stasche et al., 1997). Sialic acid deficiency and hyposialylation of glycoproteins and glycolipids appear to be a major underlying defect in GNE myopathy. Consistent with this hypothesis, a murine disease model for GNE myopathy shows clinical improvement of muscle strength and function upon metabolite supplementation of sialic acid or its precursor *N*-acetylmannosamine (ManNAc) (Malicdan et al., 2009). Notably in this context, monomeric sialic acids are decreased in quadriceps muscle of normal elder males (Marini et al., 2014). Although GNE myopathy patients can benefit from metabolic precursor supplementation (reviewed by Nishino et al., 2014), it cannot be excluded that GNE myopathy is caused by additional pathomechanisms (Krause et al., 2005; Wang et al., 2006; Amsili et al., 2008).

In a GNE myopathy patient myoblast culture model, a primary defect in response to apoptotic stimuli was observed, in particular an extended stabilization of pAkt expression (Amsili et al., 2007). Moreover, in age-related muscle deterioration a critical role for Akt has also been demonstrated (reviewed by Glass, 2003; Schiaffino and Mammucari, 2011).

Disturbance of apoptotic signaling was further supported by proteomic profiling of GNE myopathy muscle biopsy (Sela et al., 2011). In line with the hypothesis that alterations in mitochondrial metabolism might be a primary event in the pathogenetic cascade of GNE myopathy, mitochondrial deregulation was suggested at the transcriptome and morphological level (Eisenberg et al., 2008). It is well established that mitochondria are important regulators of apoptotic signaling and mitochondrial dysfunction can contribute to sarcopenia (reviewed in Marzetti et al., 2012; Marzetti et al., 2013). Although the data sets of individual proteins regulated in aging skeletal muscle differ substantially between individual proteomic surveys (Gelfi et al., 2006; Doran et al., 2007; O’Connell et al., 2007, 2008), the main trends of differentially expressed proteins involved in the cytoskeleton architecture, energy metabolism, contraction, cellular signaling and the stress response agree between various studies. These processes refer to general disturbances in skeletal muscle common to many myopathies and muscular dystrophies. In search of upstream events more specific to GNE myopathy, previously established transcriptomic data from 10 patients’ muscle biopsies (Eisenberg et al., 2008; Table S1 in Supplementary Material) were revisited. Differentially *upregulated* genes in GNE myopathy (vs. control samples with *p* ≤ 0.002) were re-assessed utilizing the DAVID bioinformatics enrichment tool (Huang da et al., 2009a,b). Interestingly, a significant upregulation of *HNRNPA2B1* (1.3-fold; *p* ≤ 0.002) was revealed along with additional RNA processing and transcription regulators including small nuclear ribonucleoprotein polypeptide G (*SNRNPG*), arginine/serine-rich-splicing factor 14 (*SUGP2/SFRS14*), TAR DNA binding protein (*TARDBP*), and heterogeneous nuclear ribonucleoprotein A3 (*HNRNPA3*). Although the upregulation of these RNA regulatory factors was comparably small, their impact on myocellular metabolism might be pathophysiologically meaningful.

Moreover, Activin A receptor, type II (*ACVR2*) was among the most significantly downregulated genes (−0.88-fold; *p* ≤ 0.002) in GNE myopathy (Eisenberg et al., 2008; Table S1 in Supplementary Material). Activins are the most efficient negative regulators of muscle mass (Chen et al., 2014). It remains to be determined whether reduced *ACVR2* expression might represent a compensatory mechanism in GNE myopathy to escape or decrease loss of muscle mass.

Finally, re-evaluation by functional annotation clustering of *downregulated* genes in GNE myopathy (Eisenberg et al., 2008) with the DAVID bioinformatics resources revealed slightly reduced expression of nuclear or steroid hormone receptors including hepatocyte nuclear factor 4-gamma (*HNF4G*), nuclear receptor subfamily 1, group I, member 3 (*NR1I3*), and nuclear receptor subfamily 1, group H, member 2 (*NR1H2*). Also, vitamin D receptor (VDR) belongs to the large family of steroid hormone receptors. Intriguingly, VDR signaling has been implicated in the regulation of calcium homeostasis, myoblast proliferation, and differentiation and might be a future approach for treatment of sarcopenia (reviewed in Wagatsuma and Sakuma, 2014). It cannot be excluded that deregulation of other components of the nuclear or steroid hormone pathway can contribute to muscle weakness and wasting in GNE myopathy.

## Multisystem Proteinopathy Associated with hnRNPA1 and hnRNPA2B1 Mutations

Autosomal-dominant mutations in the genes for the heterogeneous nuclear ribonucleoproteins *HNRNPA1* or *HNRNPA2B1* are rare causes for multisystem proteinopathy (Kim et al., 2013a). HnRNPA1 and hnRNPA2B1 are multifunctional RNA-binding proteins involved in the regulation of RNA biogenesis. The clinical phenotype may present as IBM and may be initially indistinguishable from the VCP/p97-related neuromuscular syndromes. The identified missense mutations are predicted to generate hyperstable multimers by their so-called “prion-like” domains (PrLDs), facilitate recruitment to stress granules, and drive cytoplasmic aggregate formation (Shorter and Taylor, 2013). Interestingly, also in sporadic IBM and VCP-associated myopathy, the subcellular distribution of wild type HNRNPs is altered in skeletal muscle suggesting disturbances in RNA metabolism (Pinkus et al., 2014) that might be a secondary event downstream of inflammation or protein dyshomeostasis. In multisystem proteinopathy, it remains to be determined whether hnRNPA1 or hnRNPA2B1 mutants (i) may form immediately cytotoxic oligomers, (ii) overload the proteolytic capacity of the cell, or (iii) sequester other essential proteins in cytoplasmic and nuclear aggregates. At the molecular level, also additional disease mechanisms are conceivable that may be reminiscent of sarcopenia and relate to age-dependent alterations in skeletal muscle. A well-established biomarker for cellular aging is the length of the protective caps at the physical ends of eukaryotic chromosomes, called telomeres, which shorten with each cell division cycle and with increasing chronological age. Telomerase activity is inhibited by large non-coding RNA referred to as telomeric repeat containing RNA (TERRA), which is transcribed from telomeres. Recent evidence suggests that balanced levels of hnRNPA1 and TERRA are required to regulate telomerase activity (Redon et al., 2013). This finding supports the idea that hnRNPA1 mutants might also disturb telomere formation and maintenance thereby contributing to premature aging and possibly sarcopenia.

Cellular senescence was initially defined as permanent growth arrest of primary human cells after repeated serial passaging *in vitro* (Hayflick and Moorhead, 1961). Cellular senescence is not only a safeguard against cancer but also of multifunctional physiological relevance in embryonic development, tissue repair, and aging. Novel discoveries support the hypothesis that senescence can be a highly dynamic, multi-step process (reviewed by van Deursen, 2014). Recent evidence demonstrated a close link between cellular senescence and age-dependent tissue deterioration (Baker et al., 2008). Aging increases CCN1/CYR61 expression leading to muscle senescence (Du et al., 2014). CCN1/CYR61 depends on exon skipping to provide functional protein (Hirschfeld et al., 2009). The matricellular protein CCN1/CYR61 contains several possible binding motifs (YAGR) in the exon 3–intron 3–exon 4 system for the transcription factor hnRNPA1. Disturbed or lacking HNRNPA1 due to sequestration in aggregates as suggested in multisystem proteinopathy might promote exon 3 inclusion, resulting in non-functional protein that might compromise muscle angiogenesis and endothelial cell survival (Leu et al., 2002).

## Correlation with Genetic Susceptibility of Sarcopenia and Age-Related Gene Expression in Skeletal Muscle

Hereditary IBM syndromes might involve cellular mechanisms previously related to sarcopenia and aging. Therefore, several representative susceptibility genes for sarcopenia (reviewed in Garatachea and Lucia, 2013) were evaluated to elucidate potential genetic correlations with hereditary IBMs.

A certain polymorphism in the *ACTN3* gene (R577X, rs1815739) is a well-established marker of a muscular endurance phenotype in humans. The precise localization of α-actinin 1 and GNE in the myofibrillar apparatus centered on the *Z* line remains elusive (Amsili et al., 2008). It is conceivable that physiological interaction by GNE with other resident components of the sarcomeric *Z*-disk might modulate cytoskeletal architecture and functions. Interestingly, GNE showed predominant protein expression in type II fibers in transversal muscle sections (Krause et al., 2007) and α-actinin 3 is also exclusively detected in fast-twitch (type II) fibers (Mills et al., 2001).

The myostatin phenotype is among the most favorable candidates to clarify variance among muscle phenotypes in the elder population (Garatachea and Lucia, 2013). Accordingly, also the downregulation of myostatin receptors can modulate myostatin signaling. As suggested in GNE myopathy, this mechanism could prevent muscle degeneration and might be considered an adaptive muscular response.

Epigenetic studies revealed that the differentially methylated regions related to aging are significantly enriched for muscle biogenesis (Ong and Holbrook, 2014). Similarly, VCP/p97 can regulate dynamics and chromatin organization by monoubiquitylation of histone H2B (Bonizec et al., 2015) and might contribute to pathogenic gene expression in IBMPFD.

## Conclusion

In conclusion, it will be essential to continue studying fundamental cellular pathways underlying muscular hypertrophy and atrophy to advance the discovery of promising targets for the development of causative and safe therapies for skeletal muscle disorders.

An encouraging approach is a novel strategy to promote muscle maintenance and delay muscular atrophy by utilizing an antibody, which modulates the activin type II receptor (ActRII) response (Lach-Trifilieff et al., 2014). The wide therapeutic spectrum holds promise to treat a variety of progressive neuromuscular conditions regardless of the underlying molecular defect. Another example of a non-disease specific treatment option is the molecular chaperone 4-phenylbutyrate (4-PBA), an FDA-approved substance to treat children suffering from urea cycle disorders. 4-PBA acts as an ER stress inhibitor by aiding in protein folding and preventing misfolded protein accumulation and aggregation. Recently, convincing evidence was provided that 4-PBA might be also functional to resolve protein aggregates *in vitro* and *in vivo* and to improve grip strength in a mouse model for plectinopathy, a hereditary protein aggregate myopathy (Winter et al., 2014).

In summary, recent history of gene identification in hereditary inclusion body myopathies has fostered enthusiasm to facilitate detailed understanding of molecular disease mechanisms in these familial disorders. However, the involved genes show an unprecedented functional diversity.

Therefore, a plethora of key mechanisms underlying disease onset in hereditary IBMs remain to be elucidated at the molecular and physiological level, some of which may be also relevant for the etiology of sarcopenia. Neglected aspects that may be specific to the discussed hIBMs include regulation of RNA transcription and processing, cellular senescence, angiogenesis, and *Z*-disk architecture.

Understanding the deleterious combination of disease mechanisms in detail will be an important goal for future research to establish targeted intervention strategies and to prevent sarcopenia in those at risk to develop disease-associated or age-related muscle loss. Additionally and regardless of the underlying defect, it will be important for affected patients to immediately translate current broad understanding of muscle wasting and general advances to improve muscle function into safe, approved therapy. Clinically, meaningful improvements for patients suffering from sarcopenia, hereditary, or degenerative myopathies will be the challenging goal for the immediate future.

## Conflict of Interest Statement

The author declares that the research was conducted in the absence of any commercial or financial relationships that could be construed as a potential conflict of interest.
